# Palestine 100 years ago seen through the malaria lens: an examination of successful malaria elimination, and of where the malaria community seems to have taken a wrong turning

**DOI:** 10.5281/zenodo.10974722

**Published:** 2024-04-15

**Authors:** Anton Alexander

**Affiliations:** 1 BC Business Centrum, Elscot House, Arcadia Avenue, London N3 2JU, United Kingdom.

## Abstract

It is acknowledged there exists a trend pointing to a general failure to reduce the global incidence of malaria, and the world watches anxiously in the knowledge resistance to insecticides and drugs has been developing and will intensify. Anton Alexander attempts to remind the malaria community of a successful malaria elimination that began over one hundred years ago, and by examination of that malaria elimination, explain why current anti-malaria campaigns may be considered ineffective. Alexander has conducted historical research into the first start anywhere of a successful national malaria elimination campaign over 100 years ago, and in respect to which his research papers have been published by the MalariaWorld Journal, American Entomologist and Oxford University Press.

I have been informed that Dr Pedro Alonso, former Director of the WHO Global Malaria Programme, commented: ‘Taken together, we are facing a malaria crisis. We need to do things differently. As Einstein famously said, doing the same thing over and over again and expecting a different result is insanity’ [1].

Having conducted historical research for many years into the first start anywhere of a successful national malaria elimination campaign (it first began in Palestine in 1922), I have long been puzzled why the malaria community either remains unaware of, or has chosen to ignore, the successful malaria elimination methods and the lessons learned in Palestine all those years ago, especially given the fact that malaria prevalence there was so high and caused by *Plasmodium falciparum*. In many ways similar to malaria in Africa today.

It would be harsh to describe the approach of the malaria community in its dealing with the disease as insanity, but the community’s approach does in fact appear somewhat primitively simple, in that the default position, the starting point, almost religiously, is usually based on different types of bed-nets and the chemicals with which they are treated.

The pointlessness of using bednets in dealing with malaria was clearly demonstrated in 1918 in the final year of WWI on the Palestine battlefront when the British Army decisively defeated the Turkish Army. I will not recount in detail the event here but would invite the reader to see my published paper on this event [2]. The Palestine battlefront was the only one of all the malarious battlefronts during WWI where the disease was successfully controlled.

It must be stressed that neither the 1918 successful malaria control operation nor the 1922 successful malaria elimination campaign, both in Palestine, relied upon bednets [3].

Helpfully, the World Health Organization (WHO) Handbook on Integrated Vector Management (IVM) includes a brief historical note about malaria elimination. It merely states:

*‘Before the Second World War, vector control was conducted predominantly by environmental control of the proliferation of mosquitoes.* [e.g., the 1922 Palestine method]*. .... There is evidence that environmental management had a clear impact on disease; however, elimination of disease was never on the agenda. The advent of DDT and other organochlorine pesticides during the 1940s changed this situation. ... The focus of vector control on insecticides meant that environmental management and other alternative methods were underexploited or even **forgotten**.’* [4]

The reader firstly needs to appreciate the extent and severity of the disease that existed in Palestine over 100 years ago, to give weight to the successful pioneering achievements which eventually resulted in its elimination. The reader must not be permitted to dismiss the severity as something trivial or that reports of any success against the disease are overdone or exaggerated. In 1919, Dr. Manson-Bahr, a future director of the London School of Hygiene and Tropical Medicine, described **Palestine as one of the most highly malarious countries in the world** [5]. He knew the conditions in Palestine in World War One (WWI) as an officer with the British Army that had fought and defeated the Turkish Army in Palestine in 1918. He had witnessed a British Army force in Palestine of 40,500 men lose 20,427 men in 9 weeks due to malaria. Of the 100,000 Turkish prisoners-of-war taken after their defeat in 1918 by the British Army in Palestine, 20 per cent had to be hospitalised immediately, suffering from malaria. The severity of malaria was such that had the disease not been eliminated there, it is unlikely the State of Israel (formerly Palestine until 1948) could ever have come into existence. At the time of WW1, Palestine had been drenched in malaria for generations, and the severity of the disease had rendered the country in many areas uninhabitable, desolate, almost empty (Figure 1).

**Figure 1.**
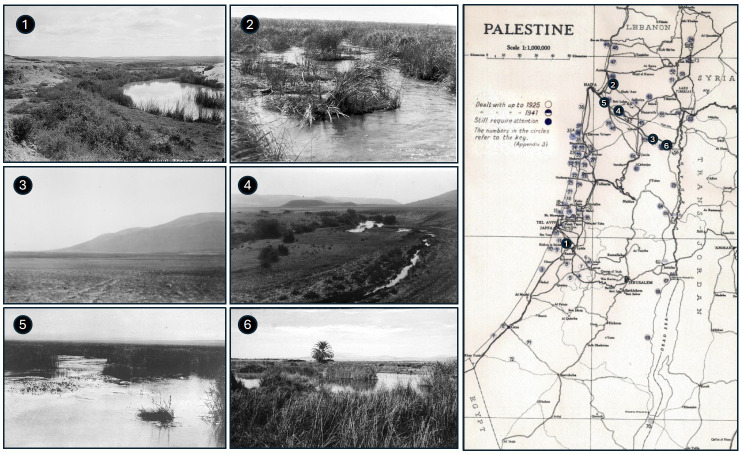
The Palestine Department of Health [7] map of the more important swamp areas in the early 1920s. Examples of areas desolate due to malaria at that time: 1. Wadi Rubin; 2. Swamps near Kurdani, south of Acre; 3. Mount Gilboa, Jezreel Valley; 4. Swamps near Kfar

Until 1922, malaria elimination was unknown; it had not been considered anywhere [3]. Forty-five years later, in 1967, WHO declared Israel malaria-free. Prior to 1922, all previous successful activities involving malaria had been only with malaria control in mind and even then, only in limited areas. US Army Surgeon-General Gorgas had employed malaria control only to enable construction in the Panama Canal Zone, the zone being only 10 miles wide and 40 miles long. General Allenby, commander of the British Army in Palestine 1917-1918 during WWI in the campaign against the Turkish Army, had employed malaria control solely to protect his troops against the disease for 6 months prior to 19th September 1918 when his final advance commenced. He defeated the Turkish Army within the next 11 days, but subsequently over 20,000 of his men (over half his army) were to die or collapse from malaria after the 11-day-incubation period, once they had left Allenby’s ‘healthy protected area’ on the 19th September, and advanced further into the remaining untreated malarious parts of Palestine previously held by the Turkish Army.

Basic anti-larval principles of malaria ***control*** had previously been applied at both the Panama Canal and in Allenby’s Palestine in 1918. But the later 1922 Palestine malaria ***elimination*** campaign contained an extra component to that of Allenby’s 1918 approach. The first component of the 1922 elimination campaign, as was the case also with Allenby’s approach, had been treatment of malaria-control as a **priority** by all involved. Malaria-control had to succeed, and failure could not be contemplated. The second component, also as with Allenby, was the **thoroughness** with which the anti-larval malaria activities had to be conducted, whilst dealing with the anti-larval measures at all times as a local problem. But the third component which was new, and was absent from Allenby’s approach, was the maintenance of the anti-larval measures, and which was to lead to eventual malaria-elimination. This third component was achieved thanks to the **education** of the few inhabitants at that time in Palestine in the early years, and which had ensured sustainable continuous malaria-control. These components, priority, thoroughness and education are rarely, if at all, achieved by the malaria community today.

## How Palestine became successful

Firstly, malaria in Palestine in 1922 was treated as a local problem, as was Allenby’s approach, with thorough destruction of all mosquito breeding sites in order to prevent or control malaria in a locality. It was conducted without reliance on mosquito bednets, drugs or insecticidal sprays for indoor use (DDT had not been (re)discovered yet).

Secondly, the next step was to take the earlier Allenby malaria control method forward towards the stage of eventual malaria elimination. Malaria elimination could be achieved if the inhabitants assisted for long enough over the years. If malaria control could be sustained for long enough, malaria elimination would be the outcome, and this is what the inhabitants had to understand and appreciate. The educational aspect of the work was certainly as important, if not more so, as any other. Once the breeding site(s) in that locality had initially been thoroughly destroyed, the inhabitants —few as there were then—through continuous, thorough and systematic larval source management, were relied upon to ensure those breeding site(s) remained unproductive for mosquitoes for years on end.

For the anti-malaria work to be effective, all maintenance had to be continuously carried out over many years, and this could only be achieved if locals understood and appreciated why they were performing the tasks. The education was successful because all inhabitants (Arabs and Jews) cooperated. They were treated with dignity and respect, every inhabitant counted, and the education that was provided ensured that if a single inhabitant had failed to understand the disease or why thorough maintenance of the destroyed mosquito breeding sites was necessary, the lesson or demonstration would be repeated. The maintenance work was often tedious, but it eventually became a routine, and due to effective education, for years the inhabitants provided their voluntary co-operation in the discharge of their role in the elimination effort.

It is worth noting that it was only once destruction of a site had successfully (i.e., thoroughly) been carried out, only then could the area be turned over to the existing or future inhabitants (who would have already been educated/trained) and who would then assume responsibility for maintaining the anti-malaria works.

In the early years, with so few inhabitants, the task of implementing these arrangements was made that much easier, and very soon the work was extended to cover the whole country.

The importance of education in respect to malaria-elimination cannot be overstated. As a result of the education, the emphasis on thoroughness was understood by the inhabitants and was accordingly maintained throughout. By 1967, the WHO had confirmed malaria had been eliminated.

## Current ineffective anti-malaria methods

The chatter now is of the Global Malaria Eradication campaign stalling, with the malaria community providing a multitude of explanations why the campaign is faltering, but much of the immediate present attention has been focused on the long-lasting insecticide-treated bednets which have sometimes been considered ineffective. The Palestine experience can provide observations or suggestions why methods at present in use around the world haven’t been effective, and the Palestine experience wouldn’t just be blaming the use of bednets. But it would be a start.

It would be good to remind why malaria in Palestine was treated as a local problem. This was due to the fact that mosquitoes travelled only a limited distance from their breeding sites in order to bite and feed on humans. If that mosquito had previously been infected, when it bit, it also injected the malaria parasite at that moment, thereby transmitting the disease. Therefore, destruction of mosquito breeding sites could have prevented or controlled malaria in that locality.

All activities involved in both Allenby’s 1918 malaria control and the subsequent 1922 Palestine malaria elimination were conducted with an emphasis on thoroughness, and, also importantly, under the direction of an entomologist. Both Allenby’s 1918 malaria control and Palestine’s national malaria elimination activities were anti-larval and were treated as local problems in respect to the mosquito breeding sites. Destruction of the mosquito breeding sites was dealt with thoroughly together with an equal emphasis on continuous attention in maintaining that destruction, ensuring these breeding sites remained destroyed. Malaria is known to be unforgiving and takes advantage of the smallest crack in anti-malaria defences. Therefore, if a breeding site has not been thoroughly and completely destroyed, or the breeding site does not remain destroyed at all times, malaria is likely to return and remain the scourge it is today.

Palestine’s success was not based on use of bed-nets but upon the thorough destruction of local mosquito breeding sites. Such destruction was visual, it could be seen, it could be physically inspected and checked, and the results would therefore be reliable, thereby enabling effective management. Today’s anti-malaria campaigns, on the other hand, with reliance on mosquito bednets, are likely to be incomplete as checks on the proper use of the nets can only be based on trust. These checks are completely dependent upon an inhabitant’s truthful and reliable account of how the net was used the previous night. It is unclear, therefore, how results and data from use of bednets can be effectively gauged and managed if the reliability and accuracy of the results and data can always be questioned. Fundamentally, correct bednet use cannot be confdently verified accurately or relied upon for scientific purposes.

Possibly the most striking practical difference between current methods and those of 100 years ago is the apparent absence today of an emphasis on thoroughness in destruction in a specified area of the mosquito or its breeding site. Thoroughness appears to have been discarded. It is missing. With the current approach, there is now no attempt to rid an area so that it is completely free of the mosquito and there is accordingly no area to be kept free. It isn’t clear how logically that situation can then ever lead to malaria elimination.

Even the USAID programmes, through their subsequent evaluations, suggested there is something missing from the programmes which appear to be trying to create a habit of a correct nightly use of bednets through awareness of the disease, but the evaluations also explained why such ‘awareness’ alone was insufficient [6].

Because malaria was treated in 1922 Palestine as a local problem, entomological skills were usually employed to identify and then destroy mosquito breeding sites, thus providing a degree of precision in the site-destruction in each locality. Due to the education and resulting cooperation, the local inhabitants over the years would then have maintained the anti-malaria works to ensure the breeding sites remained destroyed. The Palestine method was dependent upon local collective and cooperative (Jews and Arabs) assistance maintaining local anti-malaria works, but each inhabitant would have still received individual education and all inhabitants would have appreciated why the maintenance was necessary. A further point to remember, all maintenance would have been visible for inspection afterwards to ensure it was up to a required standard.

Today’s methods, however, are usually not locally based and instead have tended to be more in the nature of attempts at malaria control through use of insecticide-treated bednets and indoor residual-spraying, but these methods in recent years have met with disappointing progress which has slowed and stalled. The method is usually dependent upon each individual inhabitant using a net and accepting their houses to be sprayed, and any inspector is completely reliant on an account provided by each inhabitant as to how a net was used the previous night.

Monitoring the success or progress of attempts at malaria elimination is of the utmost importance, and without a reliable and accurate monitoring procedure, it is almost akin to 'flying blind’. Taking to the air with a blind pilot and without any wireless, controls or instruments can be likened to a suicide flight. The success or progress of the Palestine method was always going to be relatively straightforward to monitor as it contained two advantages over today’s malaria methods. Firstly, the malaria works were visible, and could be physically inspected and checked to ascertain if they were adequate or had been properly maintained. Secondly, the location of the source of new cases could be more readily identified because malaria was treated as a local problem which could be traced back and was dealt with at a local level.

Monitoring today’s malaria elimination methods appears to be a more difficult task and the approach feels incomplete or only partially effective. Today’s malaria methods usually are applied over extensive areas including differing backgrounds, localities and situations. Such areas therefore include many variations all grouped together. Most national malaria control programmes in these areas today employ verifiable indicators which monitor malaria prevalence over time or monitor malaria incidence. But it is likely such indicators will also need to display data showing how use of insecticide-treated bednets over the whole monitored area has interacted with the malaria prevalence. If it is decided a revealed trend is unsatisfactory, the diffculty with today’s malaria method is identifying ‘how, what or where is the problem’. It is then that a solution is sought for correcting or modifying the unsatisfactory trend. There are many aspects at that stage which could be affecting the trend, but finding that ‘how, what or where is the problem’ in the first place can be very difficult. It is also often easily forgotten or overlooked that part of the data upon which the unsatisfactory trend has been based may not have been monitored or verified independently and which data may have been based on an oral account of the inhabitants that may, or may not be reliable. If the involved inhabitants were educated to a standard that would have been thought acceptable in Palestine all those years ago, this latter point, namely the reliability of the oral account, would not have been so questionable.

## Conclusions

From these historical observations it can be concluded that bednets or IRS are not solutions to the malaria crisis. Their often singular blanket use will not be sufficient to drive malaria down to zero anywhere in Africa.

Practically, whilst awaiting the ‘silver bullet’ from the research laboratories and universities, there is also a need for more entomologists to project-manage, to educate and to guide with precision those involved with destruction of the mosquito breeding sites and larval source management in general.

The task of rescuing from the malaria crisis is huge, principally because the malaria community is now so set in its ways and routines. Change may not be popular within the malaria community. But change is necessary and the Palestine examples worked. Today’s anti-malaria methods don’t.

Finally, treating malaria elimination as a priority means approaching elimination of the disease as the only outcome. No other outcome can be acceptable. This is a topic that many, at first, find difficult to appreciate. Malaria elimination is not merely some mechanical process. It requires all inhabitants to be involved, to not only understand the disease itself but to also understand why it is necessary to cooperate with all instructions given for steps to eliminate the disease. This is a big commitment not only in terms of education and time but also of energy and enthusiasm, and above all, the need to succeed in eliminating the disease. That commitment is the priority.

Allenby’s 1918 malaria control and the 1922 malaria elimination campaign were conducted as a priority. Such priority came from the top, from the leadership, and for that reason, this article should be targeted at governments and other leaderships responsible for funding such malaria management. Attempts to motivate only those involved further down the management hierarchy have met with little success. History has shown that a sense of priority running down all levels throughout the whole management-hierarchy is likely to be achieved only when coming from the top.

Without priority, efforts to eliminate malaria are likely to be fruitless and pointless. Without priority, often these efforts are patronisingly referred to as ‘good works’ but in fact usually tend to be ineffective. Experience has shown, if malaria-elimination is important enough to the leadership, if it is a priority at the top, the leadership will find a way. If not a priority, it will find an excuse.

## References

[1] Alonso P Letter to malaria partners.. https://tinyurl.com/yshd9xby.

[2] Alexander A How malaria was ‘weaponised’ by the British Army during World War I.. MalariaWorld J.,.

[3] Dunkel FV, Alexander A (2020). Three stepping stones leading to malaria elimination, changing world maps on the way.. MalariaWorld J..

[4] World Health Organization: Handbook for Integrated Vector Management. Geneva, (2012). https://tinyurl.com/mryuzkxr.

[5] Austen EE (1919). Anti-mosquito measures in Palestine during the campaigns of 1917–1918.. Trans. R. Soc. Trop. Med. Hyg..

[6] USAID/Tanzania: COMMIT – Project Performance Evaluation, (2012). https://tinyurl.com/2c6tz9zn.

[7] Palestine Department of Health: A review of the control of malaria in Palestine (1918–1941). (1941). Government Printing Press, Jerusalem,. https://tinyurl.com/33btc267.

